# Lysosomal storage disorders identified in adult population from India: Experience of a tertiary genetic centre and review of literature

**DOI:** 10.1002/jmd2.12407

**Published:** 2024-01-02

**Authors:** Jayesh Sheth, Aadhira Nair, Riddhi Bhavsar, Koumudi Godbole, Chaitanya Datar, Sheela Nampoothiri, Inusha Panigrahi, Heli Shah, Shruti Bajaj, Naresh Tayade, Naveen Bhardwaj, Harsh Sheth

**Affiliations:** ^1^ Department of Molecular and Biochemical Genetics FRIGE's Institute of Human Genetics Ahmedabad India; ^2^ Department of Clinical Genetics Deenanath Mangeshkar Hospital & Research Centre Pune India; ^3^ Department of Clincial Genetics Bharati Hospital and Research Centre Pune India; ^4^ Department of Paediatrics Amrita School of Medicine Kochi India; ^5^ Department of Pediatrics Postgraduate Institute of Medical Education and Research, PGIMER Chandigarh India; ^6^ Department of Pediatrics Smt. NHL Municipal Medical College Ahmedabad India; ^7^ The Purple Gene Clinic Mumbai India; ^8^ Department of Pediatrics Dr. Panjabrao Deshmukh Memorial Medical College Amravati India; ^9^ Department of Pediatrics AIIMS Hospital Bhatinda Punjab India

**Keywords:** adult‐onset LSDs, Fabry disease, Gaucher disease, p.Leu483pro

## Abstract

Lysosomal storage disorders (LSDs) in adults have milder phenotype and variable age at presentation. Several studies have described the phenotype, genotype and treatment outcomes for adult‐onset LSDs like Gaucher, Fabry, Pompe disease and others. We describe the first systematic study on the occurrence of LSDs in an adult population from India. It describes, the key clinical signs seen in these patients and those from literature review that can aid in early detection. Of 2102 biochemically diagnosed LSDs cases, 32 adult patients were identified with LSDs. Based on the clinical suspicion, screening test and enzyme study was carried out. Twenty‐two patients were subjected to a genetic study to identify the causative variant in a respective gene. Of the 32 adult patients, we observed a maximum percentage of 37.5% (*n* = 12) cases with Gaucher disease, followed by 13% (*n* = 4) with Fabry disease. We found 10% of cases with MPS IVA and MPS I, and 9% cases with Pompe. Single case of adult mucolipidosis III and two cases each of Type 1 Sialidosis, Niemann‐Pick disease B and metachromatic leukodystrophy were identified. We observed two common variants p.Leu483Pro and p.Ala487Thr in the *GBA1* gene in 23% of Indian patients with adult Gaucher disease. No common variants were observed in other aforementioned LSDs. Study identified 50% of Fabry patients and 4% of Gaucher patients diagnosed at our centre to be adults. The prevalence of adult Pompe patients was low (3.4%) as compared to 80% reported in the Caucasian population. Adult LSDs such as, MPS III, GM1/GM2 gangliosidosis and Krabbe disease were not identified in our cohort.

**Synopsis**

Overall, study from our cohort highlights Gaucher and Fabry disease to be among the common LSDs in the adult population in India, which is in concordance with reports by other groups. However, common variants previously reported in adult Gaucher and Fabry patients were not identified in our cohort, suggesting global genetic heterogeneity. The clinical pointer in this group were found to be unexplained mild hepatosplenomegaly, unexplained avascular necrosis of pelvic bone, angiokeratoma, muscle weakness, short stature with skeletal dysplasia, gait imbalance with tremors, myoclonus and cherry red spot. However, many adult LSDs are missed due to subtle clinical presentation and, awareness among adult neurologists, dermatologists and psychiatrists is necessary for their timely detection.

## 1 INTRODUCTION

Lysosomal storage disorders (LSDs) are a group of more than 70 inherited diseases due to defects in genes encoding lysosomal hydrolases and proteins. They have a combined incidence of 1 in 5000 live births.^1^ The age of onset of symptoms is variable based on which, LSDs are classified into infantile, juvenile, and adult‐onset types. Patients with juvenile‐onset generally are normal up to the age of 2–5 years followed by gradual expression of symptoms. In adult‐onset patients, the symptoms appear in the late teens or adulthood or the early symptoms are mild.^1^

The proportion of adult cases reported for LSDs namely Gaucher disease, Fabry disease and Pompe disease is ~13%, 85% and 65%, respectively^2^, ^3^, ^4^ in their corresponding global disease registries. However, their true incidence is not known. Furthermore, very few reports of adult LSDs like Tay–Sachs disease, Sandhoff disease, GM1 gangliosidosis have been described in the literature.^5^, ^6^, ^7^ A diagnostic delay is reported for LSDs due to presence of overlapping clinical phenotype and variable degree of severity.^8^ Of note, the reported mean delay is ~15 years from symptom onset to a diagnosis in adult LSD cases.^9^ This delay suggests that there is poor awareness of this group of LSDs among the medical professionals seeing cases beyond the paediatric age group. Nonetheless, due to availability of treatment options for some LSDs,^10^ increasing studies are being directed towards understanding the epidemiology and pathophysiology of adult patients with LSDs in the last decade.^11^, ^12^, ^13^ Additionally, recent studies have also shown a relationship between certain LSD gene variants and neurodegenerative disorders like Parkinson disease in adults.^14^ Hence, studies aimed at identifying the prevalence and burden of this group in different populations is necessary.

At present, there is no epidemiological data available for LSDs in the adult population in India. Previously, few cases of adult LSDs namely Gaucher disease, Fabry disease, Pompe disease, GM1 gangliosidosis and Sialidosis have been reported by different groups across India.^15^, ^16^, ^17^ The present study describes the experience of adult cases of LSDs from a tertiary Genetic centre in India. We also explore their burden and the potential clinical symptoms that can aid in early detection of these cases. Additionally, we have performed review of cases of adult with LSDs in the literature to outline the key clinical signs in these conditions and to assess genotype–phenotype correlations.

## 2 MATERIALS AND METHODS

### 2.1 Patients

The present study comprised of 32 adult patients referred by clinicians from across India with a clinical suspicion of LSDs. These cases were pooled in a span of 15 years (2008–2023) from a total of 4562 cases suspected with LSDs. Out of these, 2102 cases were enzymatically diagnosed with a particular LSD (46.1%), of which ~1.4% constituted the adult patients. This study cohort included 19 males and 13 females in the age range of 18–56 years at the time of the investigation. Informed consent for the investigations was obtained from all patients. The Institutional ethics committee at FRIGE's Institute of Human Genetics approved this study in accordance with the Helsinki declaration (Reg No‐E/13237).

### 2.2 Biochemical investigations

A peripheral blood sample in EDTA vial was collected from all patients and additionally, a urine sample was collected in cases with clinical suspicion of mucopolysaccharidosis (MPS). For cases with a preliminary suspicion of Gaucher disease, plasma chitotriosidase levels were assessed.^18^ In cases suspected of MPS, urine glycosaminoglycans were tested, both quantitatively and qualitatively as described previously.^19^ An I‐cell screening test using a plasma sample was performed for the case with suspicion of mucolipidosis.^20^

### 2.3 Enzyme assay from leukocytes

Leukocytes were isolated from peripheral whole blood and subjected to the standard protocol for enzyme assay using 4‐MU fluorometric assay or PNCS spectrophotometric synthetic substrate as shown by Sheth et al. 2014, Van Diggelen et al. 1990 and Lee‐Vaupel et al. 1987.^21^, ^22^, ^23^

### 2.4 Total and free *N*‐acetyl neuraminic acid estimation from urine

Urine samples of the patient were subjected to treatment using the protocol described by Waters et al. 1992^24^ for estimation of total and free *N*‐acetyl neuraminic acid (NANA).

### 2.5 DNA extraction and storage

Genomic DNA was extracted from the peripheral blood of samples using the salting out technique.^25^ The genomic DNA was quantified using a QIAxpert (Cat. No: 9002340) from Qiagen, run on 2% agarose gel to check its quality and was then stored at −20°C until further investigations.

### 2.6 Preliminary testing of the p.Leu483Pro variant in the *GBA1* gene in Gaucher patients

The DNA samples of nine patients enzymatically diagnosed with Gaucher disease were amplified using primers mentioned in the Supporting Information File S1. Amplification conditions included 5‐min denaturation at 94°C followed by 30 cycles each consisting of 1 min denaturation at 94°C for 45 s of annealing at temperature 61°C, and 45‐s extension at 72°C. The PCR product was then subjected to restriction enzyme digestion using MspI (New England Biolabs). For this, 10 μL of PCR product was incubated with 0.5 μL of MspI enzyme (10 U/mL) at 37°C for 3 h. The digested product was then separated on a 2.5% agarose gel. On the basis of the size of different bands obtained, the mutation p.Arg502Cys and p.Leu483Pro can be identified. In Gaucher patients negative for p.Leu483Pro variant in the *GBA1* gene and for Sialidosis patients, Sanger sequencing was performed for exons of *GBA1* and *NEU1* gene using primers mentioned in Supporting Information File S1.

### 2.7 Single‐molecule molecular inversion probes based sequencing analysis

DNA samples of nine patients enzymatically diagnosed with a particular LSD (one case of Gaucher, three cases of Fabry, two cases of MPS‐I, one case of Niemann–Pick disease B [NPD‐B], one case of Pompe and one case of MPS‐IVA) were subjected to a targeted gene panel study. This panel was based on single molecule molecular inversion probes for 23 genes associated with common lysosomal storage diseases in India. Target genomic regions were captured in a reaction containing smMIPs and genomic DNA in a molecular ratio of 1000:1 as described previously by Hiatt et al. 2013.^26^ The resulting library was then sequenced on the Illumina MiSeq platform at a mean coverage of 200×. The obtained reads were aligned to the human reference genome assembly (GRCh37/hg19) using BWA^27^ and germline variants were called using GATK v4.1.^28^ Variants were annotated, filtered and prioritised based on the proband's phenotype (in HPO format) using Exomiser v13^29^ integrating data from SIFT, Polyphen2, MutationTaster, Combined Annotation Dependent Depletion (CADD) scores, dbSNP (www.ncbi.nlm.nih.gov/SNP/), the Genome Aggregation Database (gnomAD; gnomad.broadinstitute.org) and ClinVar (www.ncbi.nlm.nih.gov/clinvar).

## 3 RESULTS

### 3.1 Study cohort

This study describes 32 adult patients who were clinically suspected with a particular LSD and were subsequently diagnosed by a lysosomal enzyme study. A genetic study to identify the causative variant was performed in 22 out of the 32 patients. In 10 patients, a confirmatory DNA testing could not be carried out either due to the absence of patient consent or the patient was lost to follow‐up. The average age at presentation in this cohort was 27 years; 70% of the patients were between the ages of 18 and 30 years at the time of diagnosis.

### 3.2 Adult Gaucher disease

Over the span of 15 years (2008–2023), 339 cases have been diagnosed with Gaucher disease using enzyme study and/or DNA testing at our centre. We found ~4% of these cases with adult‐onset phenotype. Adult Gaucher patients comprise a maximum percentage of 37.5% (*n* = 12) in this cohort. While the precise age of onset was not known for all cases, the age at diagnosis ranged from 18 to 44 years. The first seven patients (P1–P7) have been described previously.^15^ Overall, the most common presenting clinical phenotype in these patients included hepatosplenomegaly and bone pain. Plasma chitotriosidase levels ranged from 43.5 to 72 000 nmol/h/mL plasma with patients P1, P2, P4, P5, P7, P9, P11 and P12 showing markedly elevated levels. β‐glucosidase levels were analysed from leukocyte sample and a decreased activity (less than 10%) was observed, thereby confirming the diagnosis of Gaucher disease. Table 1 describes the clinical details, enzyme activity and variant details for these patients. Following this, a genetic study was performed for 11 patients. We found a common variant c.1448T > C (p.Leu483Pro) present in three patients in a heterozygous state with another pathogenic variant on the second allele in the *GBA1* gene. Likewise, another variant c.1459G > A (p.Ala487Thr) in the *GBA1* gene was identified in three patients. Interestingly, one patient, P12, is a Gaucher disease Type 3C patient identified with the common variant p.Asp448His in the *GBA1* gene. Only 40 Gaucher disease Type 3C patients are reported in the literature with one patient previously reported from India.^30^

**TABLE 1:** Clinical details, enzyme and molecular study details for the 32 adult LSD patients.

Patient ID	Phenotype observed	Sex	Age at diagnosis (years)	Enzyme name	Enzyme activity (nmol/h/mg protein)^a^	Chitotriosidase level (nmol/h/mL plasma)	Disease	Molecular study		
								Gene	Variant (codon change/protein change)	Zygosity
P1	At age of 15 years, difficulty in walking, x‐ray: avascular necrosis of left head of femur, BM s/o foamy cytopenia with splenomegaly, HB‐6.9	M	20	β‐glucocerebrosidase	2.5	57 503.7	Gaucher disease	*GBA*	c.1448 T > C (p.L483P)	Compound heterozygous
									c.1102C > T (p.R368C)	
P2	Splenomegaly, anaemia	F	20	β‐glucocerebrosidase	1.2	72 000	Gaucher disease	*GBA*	c.1459G > A (p.A487T)	Homozygous
P3	Hepatosplenomegaly	F	26	β‐glucocerebrosidase	1.2	0	Gaucher disease	*GBA*	c.1459G > A (p.A487T)	Homozygous
P4	No clinical details	M	31	β‐glucocerebrosidase	NA	14 378	Gaucher disease	*GBA*	c.1060G > A (p.D354N)	Homozygous
P5	Hepatosplenomegaly, thrombocytopenia, BM s/o Gaucher cells	M	25	β‐glucocerebrosidase	NA	1670	Gaucher disease	*GBA*	c.1448 T > C (p.L483P)	Compound heterozygous
									c.167 T > G (p.V56G)	
P6	Splenomegaly, anaemia, Thrombocytopenia, BM s/o Gaucher cells	F	28	β‐glucocerebrosidase	NA	102.4	Gaucher disease	*GBA*	c.1459G > A (p.A487T)	Compound heterozygous
									c.492C > G (p.S125R)	
P7	H/o generalised weakness since last 2 years, moderate to severe splenomegaly, BM study consist with Gaucher cells.	F	40	β‐glucocerebrosidase	1.5	54 503.7	Gaucher disease	*GBA*	c.1300C > T (p.R434C)	Homozygous
P8	Suspected Gaucher disease assessed by liver and BM histopath, chronic anaemia, splenectomy done at 12 years of age had osteomelytis of right femur, surgical operation done, mentally normal—presently studying in polytechnique first year	M	19	β‐glucocerebrosidase	3.4	6412.5	Gaucher disease	*GBA*	c.1504C > T (R502C)	Homozygous
P9	No clinical details	F	26	β‐glucocerebrosidase	1.8	ND	Gaucher disease	*GBA*	ND	
P10	Splenomegaly, biopsy showed Gaucher cells	M	22	β‐glucocerebrosidase	2.8	43.5	Gaucher disease	*GBA*	c.1504C > T (p.R502C)	Homozygous
P11	Hepatosplenomegaly, splenectomy at 7 years, bilateral hip replacement, liver biopsy: extensive bone marrow necrosis	F	29	β‐glucocerebrosidase	1.3	26273.1	Gaucher disease	*GBA*	c.1448 T > C (p.L483P)	Compound heterozygous
									c.913C > G (p.P305A)	
P12	Anaemia, corneal opacity, oculomotor apraxia, aortic calcification	F	18	β‐glucocerebrosidase	1.5	25092.5	Gaucher disease	*GBA*	c.1342G > C (p.D448H)	Homozygous
P13	Short stature, skeletal dysplasia, x‐ray: anterior beaking of spine, spondyloepiphyseal dysplasia	M	18	β‐galactose‐6‐sulphatase	0.53	ND	MPS‐IVA	*GALNS*	ND	
P14	No clinical details	F	31	β‐galactose‐6‐sulphatase	0.8	ND	MPS‐IVA	*GALNS*	ND	
P15	No clinical details	M	30	β‐galactose‐6‐sulphatase	0.2	ND	MPS‐IVA	*GALNS*	c.230C > G (p.P77R)	Homozygous
P16	Large eyes, proptosis, MRI: leukodystrophy, ECHO: bicuspid aortic valve reduced LV compliance, short metacarpals, stiff joints	F	29	α‐iduronidase	1.9	ND	MPS‐I	*IDUA*	c.891C > A (p.N297K)	Homozygous
P17	Mild coarse face, skeletal dysplasia, stiff joints	M	28	α‐iduronidase	0.5	ND	MPS‐I	*IDUA*	ND	
P18	Coarse face, corneal clouding, depressed nasal bridge, kyphoscoliosis, mild hepatosplenomegaly, stiffness	M	18	α‐iduronidase	0	ND	MPS‐I	*IDUA*	c.1469 T > C (p.L490P)	Homozygous
P19	Acroperasis and stroke at an early age, angiokeratoma	M	46	α‐galactosidase	1.75	ND	Fabry disease	*GLA*	c.1088G > A (p.R363H)	Hemizygous
P20	Angiokeratoma	M	35	α‐galactosidase	2.88	ND	Fabry disease	*GLA*	c.25del (p.H9IfsTer112)	Hemizygous
P21	Reddish brown rash, nodular red lesion mainly involving trunk, s/o cutaneous angiokeratoma, h/o inguinal hernia at birth (repaired at 3 months)	M	30	α‐galactosidase	3.8	ND	Fabry disease	*GLA*	ND	
P22	Angiokeratoma, xerosis on back, corneal verticillata on both eyes, ECHO and USG‐ normal	M	25	α‐galactosidase	3.2	ND	Fabry disease	*GLA*	c.828C > A (p.S276R)	Hemizygous
P23	Hip dysplasia, short spine, short stature, waddling gait, joint pain, short broad hands with camptodactyly, no dysmorphism, skeletal dysplasia	M	26	Hexosaminidase‐A	24907.9	ND	ML II/III	GNPTAB/GNPTG	ND	
				Hexosaminidase‐T	55408.7					
				Arylsulfatase‐A	4012.4					
				β‐glucuronidase	10959.4					
P24	Epilepsy at 17 years of age, myoclonus, tremors, ataxia, gait disturbance, cherry red spot in fundus	M	24	Total NANA	1.2	ND	Sialidosis	*NEU1*	c.1021 + 4A > T	Homozygous
P25	Myoclonic seizures, intermittent fall while walking; improper speech (Dysarthria) and visual disturbances, long face, arachnodactyly, and look tall in stature. MRI of his brain was suggestive of mild cerebral and cerebellar atrophy.	M	31	ND	ND	ND	Sialidosis	*NEU1*	c.1055A > C (p.N352T)	Compound heterozygous
									c.727G > A (p.G243R)	
P26	Progressive proximal muscle weakness and frequent respiratory infections	F	41	α‐glucosidase (with acarbose/without acarbose)	ND	ND	Pompe disease	*GAA*	c.1841C > T (p.T614M)	Homozygous
P27	Generalised muscle weakness	M	32	α‐glucosidase (with acarbose/without acarbose)	0.19/13.2	ND	Pompe disease	*GAA*	ND	
P28	Weakness of B/L lower limbs since 3 years of age, left eyelid ptosis, h/o calf pain, weakness while climbing up and down stairs	F	18	α‐glucosidase (with acarbose/without acarbose)	0.36/1.56	ND	Pompe disease	*GAA*	ND	
P29	ataxia and bilateral tremors	M	23	Arylsulfatase‐A	0.28	ND	MLD	*ARSA*	ND	
P30	Gait ataxia, difficulty in walking, nystagmus, tremors in both hands	M	19	Arylsulfatase‐A	2.9	ND	MLD due to Sap‐B deficiency	*PSAP*	c.593G > A (p.C198Y)	Homozygous
P31	Unexplained splenomegaly	F	20	Sphingomyelinase	0.6	2786.9	Niemann‐pick disease B	*SMPD1*	ND	
P32	Hepatosplenomegaly, anaemia, thrombocytopenia, short stature, restricted lung function	F	36	Sphingomyelinase	0.9	10419.3	Niemann‐pick disease B	*SMPD1*	c.1693G > T (p.Asp565Tyr)	Homozygous

Abbreviations: LSD, lysosomal storage disorder; NA, not available; ND, not done.

^**a**^ Normal range for enzyme activity: β‐glucocerebrosidase: 4.0–32.8 nmol/h/mg protein, β‐galactose‐6‐sulphatase: 3.9–42.6 nmol/h/mg protein; α‐iduronidase: 6.1–23.9 nmol/h/mg protein, α‐galactosidase: 8.1–28.5 nmol/h/mg protein, Total NANA: 0.35 ± 0.21 nmol/g creatinine; α‐glucosidase (with acarbose/without acarbose): 3.0–21.9/9.3–51.0 nmol/h/mg protein, arylsulphatase‐A: 0.6–4.99 nmol/h/mg protein; sphingomyelinase: 1.8–9.6 nmol/17 h/mg protein, hexosaminidase‐A: 201.1–2594.6 nmol/hr/mL plasma; hexosaminidase‐T: 773.6–4686.6 nmol/h/mL plasma, arylsulfatase‐A: 34.0–268.5 nmol/h/mL plasma; β‐glucuronidase: 116.4–965.2 nmol/h/mL plasma.

### 3.3 Adult MPS disorder

**FIGURE 1:**
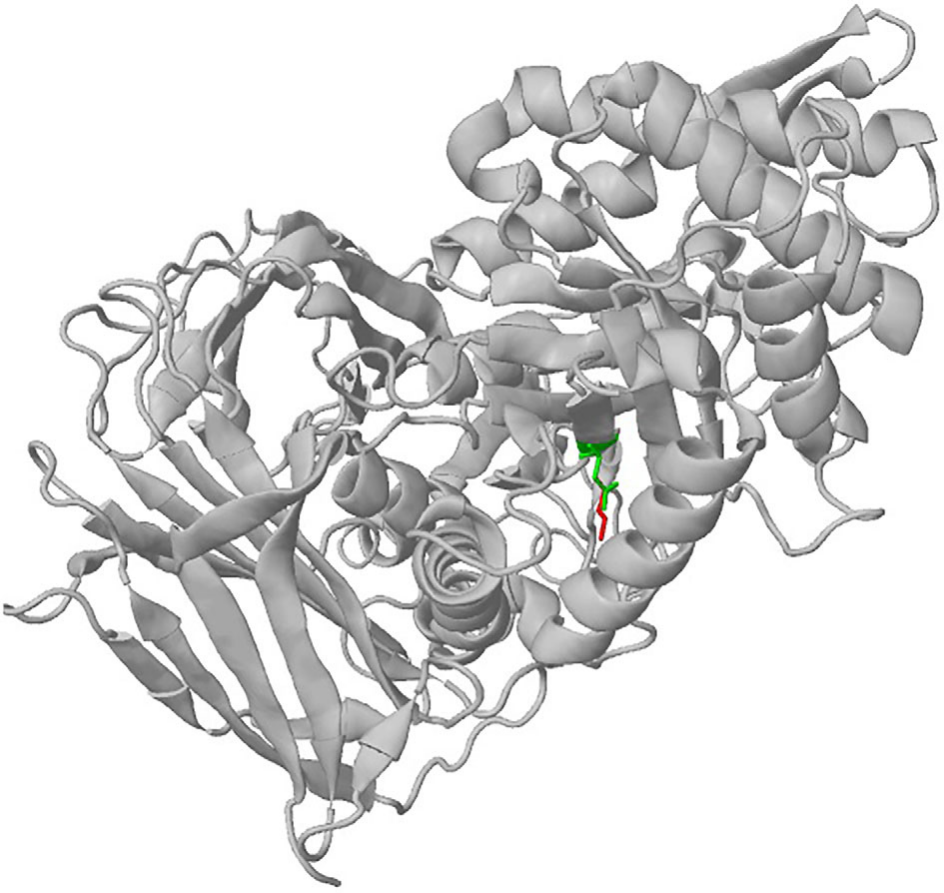
Predicted 3D structure of the IDUA protein due to the variant c.891C > A (green: wild‐type residue: Asn; red: mutant residue: Lys).

Out of 232 MPS IV A and 92 MPS I patients enzymatically and/or molecularly diagnosed at our centre between 2008 and 2023, adult cases constituted 1% (*n* = 3) and 3% (*n* = 3), respectively. For patients P13–P15 suspected with MPS IVA, urinary glycosaminoglycan (GAG) analysis showed elevated GAG excretion with excess of keratan sulphate. Subsequently, low activity of enzyme *N*‐acetylgalactosamine 6‐sulphatase in these patients confirmed the diagnosis of MPS IVA. The founder variant p.Pro77Arg in the *GALNS* gene, previously reported in the Patel community from Gujarat, India,^31^ was identified in patient P14. Patients P16–P18 suspected with MPS I, showed mild coarse face, with corneal clouding seen only in patient P17. P17 and P18 showed skeletal dysplasia and kyphoscoliosis. Remarkably, MRI report for patient P16 showed changes of leukodystrophy in bilateral deep and subcortical white matters in the fronto‐parietal occipital region, which gave a preliminary clue for adult‐onset leukodystrophy or MPS disorder. A low α‐iduronidase activity was observed in these patients (Table 1), thus confirming the diagnosis of MPS I. Additionally, a novel missense variant c.891C > A and a previously reported variant c.1469 T > C in the *IDUA* gene was identified in patients P16 and P18, respectively. Prediction of the effect of novel variant using Missense3D (http://missense3d.bc.ic.ac.uk/~missense3d/, assessed on 27 September 2023) (Figure 1) showed that the substitution replaces a buried uncharged residue (Asn) with a charged residue (Lys), which may destabilise the protein.

### 3.4 Adult Fabry disease

Overall, eight male patients have been biochemically diagnosed with Fabry disease at our centre, of which 50% (*n* = 4) were adults. The key phenotype noticed in Fabry patients P19–P22 was angiokeratoma. Corneal verticillata was seen in patient P22. All patients showed a reduced activity of α‐galactosidase (less than 10%). We identified two novel variants, c.25del (p.H9IfsTer112) and c.828C > A (p.S276R) in the *GLA* gene in patients P20 and P22, respectively. For novel missense variant p.S276R, Missense3D (http://missense3d.bc.ic.ac.uk/~missense3d/, assessed on 27 September 2023) predicted no structural damage in the resulting protein; however, substitution of a polar uncharged amino acid (serine) by a basic amino acid (arginine) is likely to affect the protein stability. A previously reported variant c.1088G > A (p.R363H) in the *GLA* gene was detected in P20.

### 3.5 Adult mucolipidosis III

We found one adult case out of 75 biochemically diagnosed mucolipidosis II/III cases at our centre between 2008 and 2023. This patient (P23) presented with clinical indications of hip dysplasia, short stature, and short broad hands with camptodactyly. No facial dysmorphism was observed; however, the patient had a waddling gait and experienced joint pain. I‐cell screening test was positive. The enzyme activity levels of hexosaminidase‐A (Hex‐A), hexosaminidase‐T (Hex‐T), arylsulphatase‐A (ARSA) and β‐glucuronidase were elevated in plasma (Table 1). This confirmed the diagnosis of Mucolipidosis III. However, as the patient was lost to follow‐up, a molecular study could not be performed.

### 3.6 Adult Sialidosis

Two cases of Sialidosis were diagnosed in (P24 and P25). The key complaints were myoclonic jerks, gait disturbance and frequent falls. Both experienced generalised tonic–clonic seizures. Brain MRI showed chronic ischemic white matter changes. For patient P25, cherry red spot in the fundus was noted. A preliminary screening test for oligosaccharidoses was positive for both of them. Increased levels of free and total NANA in the urine sample was observed in P25 (Table 1). Genetic study identified novel homozygous splice‐site variant c.1021 + 4A > T in Intron 5 of the *NEU1* gene in P25 and likely compound heterozygous variants c.1055A > C (p.N352T) and c.727G > A (p.G243R) in Exons 6 and 4 of the *NEU1* gene, respectively, in P26.

### 3.7 Adult Pompe disease

Three cases of adult Pompe patients (3.4%) were identified out of 78 Pompe patients biochemically diagnosed at our centre. Patients (P26–‐P28) presented with weakness of bilateral lower limbs and muscle myopathy were prominent features. Patient P28 had left eyelid ptosis, a history of calf pain, and experienced weakness while climbing stairs. The levels of alpha‐glucosidase in patients P27 and P28 were found to be less than 10% of the mean activity. For patient P26, we identified a previously reported variant c.1841C > T (p.Thr614Met) in the *GAA* gene. This established the diagnosis of Pompe disease; however, genetic study was not performed in other patients due to their refusal.

### 3.8 Adult metachromatic leukodystrophy

Two patients (P29 and P30) were diagnosed with adult metachromatic leukodystrophy (MLD). P29 showed decreased activity of arylsulphatase‐A enzyme; however, in patient P30, a normal activity of arylsulphatase‐A enzyme was observed (Table 1). Gait ataxia, difficulty in walking, speech and tremors in both hands were the key complaints in this patient. We identified a likely pathogenic variant c.593G > A in the *PSAP* gene,^32^ thus confirming the diagnosis of MLD due to Sap‐B deficiency in P30. For P29, a molecular study was not performed due to patient refusal.

### 3.9 Adult NPD B

Of the 175 NPD A/B cases, biochemically diagnosed at our centre between 2008 and 2023, we found two adult patients P31 and P32 (1.4%). Both patients presented with unexplained splenomegaly. A reduced activity of the acid sphingomyelinase (Table 1) confirmed the diagnosis of NPD B. P31 was lost to follow‐up and hence a molecular study could not be carried out. In P32, DNA testing revealed a previously reported variant c.1693G > T (p.Asp565Tyr) in Exon 6 of the *SMPD1* gene.

## 4 DISCUSSION

To the best of our knowledge, this study is a first of its kind that defines the presentation of LSDs in adult population from India and provides an estimate of the proportion of adults with LSDs in the country. An early diagnosis of adult LSD patients is critical to provide them with timely treatment intervention that can save them from a physical and emotional stress with financial burden. Treatment options like enzyme replacement therapy, bone marrow transplantation, substrate reduction therapy are now available for common LSDs like Gaucher disease, Pompe disease, and Fabry disease.^10^ In India too, therapy is being provided to around 300 LSD patients under various charitable programmes namely INCAP (India's charitable access programme), Shire's HGT charitable access programme and few under rare disease policy of the Government of India.^33^ Approximately, 40 of these patients are likely to be an adult now. Hence, exact number of adult LSD patients receiving therapy cannot be ascertained and to the best of our knowledge, none of the patients except one with GD and one with Fabry disease described in our study received enzyme replacement therapy.

Overall, Gaucher and Fabry disease were the common LSDs in our present adult cohort, which is similar to that observed in the world^1^ (Table 2). Interestingly, different groups have seen a high percentage of adult population (~ 90%) in case of Fabry disease.^16^ This was also the case in our centre where 50% of our Fabry patients were adults. On the other hand, the proportion of adults with Pompe disease was low (3.4%) as compared to 20.8% reported in a previous study on Pompe patients in India.^17^ Low number of adult Pompe patients in this study could be attributed to the lack of awareness among adult neurologist as these patients are often referred to them as a neuro muscular disorder.

**TABLE 2:** Clinical signs and genetic variants reported in common adult‐onset LSDs in populations across the world.

Disease name	Average age at diagnosis (years)	Common phenotype noted	Total no of patients	Gene (Transcript)	Common mutation reported (% cases with the genotype)	Country	Reference
Gaucher disease	21	Hepatosplenomegaly, thrombocytopenia, anaemia, difficulty in walking, bone pain	103	*GBA1* (NM_000157.4)	p.N370S (81%)	Israel, Germany	Dinur et al.,^34^
			7	*GBA1* (NM_000157.4)	p.A487T (43%); p.L483P (43%)	India	Sheth et al., 2018^15^
Niemann‐pick disease B	45.2	Hepatosplenomegaly, anaemia, thrombocytopenia, interstitial lung disease, low HDL	28	*SMPD1* (NM_000543.5)	p.Arg610del (61%)	France	Lidove et al., 2017^38^
			14	*SMPD1* (NM_000543.5)	p.Arg610del (64%)	Netherlands	Hollak et al., 2012^55^
Tay‐Sachs disease	18	Nonspecific cerebellar atrophy, anterior motor neuron involvement, progressive proximal muscle weakness, ataxia, gait, psychosis	21	*HEXA* (NM_000520.6)	G269S/TATC1278 (66%)	USA	Neudorfer et al., 2005^56^
			14	*HEXA* (NM_000520.6)	G269S (14 cases) (observed in compound heterozygous state with pathogenic variant on the other allele)	Czech Republic	Jahnová et al., 2018^57^
Sandhoff disease	30	Lower motor neuron phenotypes, and sometimes with amyotrophic lateral sclerosis‐like features, cerebellar atrophy	1	*HEXB* (NM_000521.4)	IVS2‐1G > A; c.1598G > A	Japan	Yoshizawa et al., 2002^58^
			1	*HEXB* (NM_000521.4)	c.619A > G; c.1250C > T	Korea	Kang et al., 2013^59^
			1	*HEXB* (NM_000521.4)	HEXB gene deletion; c.1513C > T	Philippines	Beecher et al., 2022^6^
			1	*HEXB* (NM_000521.4)	c.298delC; G473S l	USA	Sung et al., 2018^60^
			1	Not available	Not available	Lebanon	Khoueiry et al., 2019^61^
GM1 gangliosidosis	33	Gait disturbance, dystonia, parkinsonism, dysarthria, abnormal MRI	16	*GLB1* (NM_000404.4)	c.152 T > C	Japan	Yoshida et al., 1991^62^
			3	Not available	Not available	India	Muthane et al., 2004^7^
			10	Not available	Not available	Brazil	Giuglani et al., 2019^63^
			8	Not available	Not available	Brazil	Kannebley et al., 2015^64^
Fabry disease	47	Neuronopathic pain, angiokeratoma, proteinuria, renal insuficiency, cardiac symptoms	251	*GLA* (NM_000169.3)	c.644A > G (p.Asn215Ser) (33%)	UK	Lavalle et al., 2018^42^
			203	*GLA* (NM_000169.3)	c.339 T > A (p.Phe113Leu) (203 cases)	Portugal	Azevedo et al., 2020^43^
MPS‐III A	38.3	Delayed speech, hyperactivity, retinal dystrophy, left ventricular hypertrophy	13	*NAGLU* (NM_000263.3)	c.734G > A (p.Arg245His); c.545G > A (p.Arg182His)	Netherlands	Nijmeijer et al., 2019^13^
MPS‐III B	21	Neurocognitive impairment, hyperactivity	3	*NAGLU* (NM_000263.3)	c.1927C > T (p.Arg643Cys); c.1834A > G (p.Ser612Gly)	Netherlands	Nijmeijer et al., 2019^13^
MPS‐III C	44	Neurocognitive impairment, hyperactivity	2	Not available	Not available	Netherlands	Berger‐Plantinga et al., 2004^65^
MPS‐IV A	30	Short stature, Bone dysplasia, hearing loss, ligamentous laxity, odontoid dysplasia, abnormal gait	2	*GALNS* (NM_000512.4)	c.491A > C (p.Asn164Thr); c.901G > T (p.Gly301Cys)	Columbia	Erazo‐Narváez et al., 2020^66^
			33	*GALNS* (NM_000512.4)	c.1156C > T (24%)	Spain	Quijada‐Fraile et al., 2021^67^
			27	*GALNS* (NM_000512.4)	Not available	Brazil, Colombia, Germany, Spain, Turkey and the UK	Hendriksz et al., 2014^68^
Metachromatic leukodystrophy	38	Post‐partum depression, mental deterioration, behavioural abnormalities, ataxia	2	*ARSA* (NM_000487.5)	Not available	Austria	Kumperscak et al., 2005^69^
			1	*ARSA* (NM_000487.5)	L289S; T409I	Japan	Ito et al., 2008^70^
			1	*ARSA* (NM_000487.5)	c.256C > T (Arg86Trp)	Italy	Benzoni et al., 2021^71^
			16	*ARSA* (NM_000487.5)	Not available	France/Switzerland	Baumann et al., 1991^40^
			8	*ARSA* (NM_000487.5)	c.542 T > G; c.465 + 1G > A (25%)	Italy	Cesani et al., 2015^72^
			1	*ARSA* (NM_000487.5)	I179S	Tunisia	Chebel et al., 2009^52^
Krabbe disease	33	Hemiparesis, weakness in both legs, spastic gait	1	*GALC* (NM_001201401.1)	c.1901 T > C	Italy	Durães et al., 2021^73^
			6	*GALC* (NM_001201401.1)	c.1901 T > C (six cases)	China	Zhang et al., 2018^74^
			4	*GALC* (NM_001201401.1)	30 kb del (four cases)	New York	Duffner et al., 2012^75^
			1	*GALC* (NM_001201401.1)	c.865G > C (p.G289R); c.136G > T (p.D46Y)	China	Zhaung et al., 2019^76^
			7	*GALC* (NM_001201401.1)	30 kb del (42.8%)	France	Deb et al., 2013^77^
			1	*GALC* (NM_001201401.1)	p.G286D and p.Y490N	Italy	Iacano et al., 2022^78^
Neuronal ceroid lipofuscinosis (Autosomal dominant kuff's disease)	35	Seizures, dementia, myoclonus, epilepsy, abnormal EEG	11	*DNAJC5* (NM_025219.3)	c.343_345 CTC; p.Leu115Arg (11 cases)	USA	Boehme et al., 1971^79^
			10	*DNAJC5* (NM_025219.3)	Not available	USA	Josephson et al., 2001^80^
			1	*DNAJC5* (NM_025219.3)	c.370_399dup	Czech Republic	Jedličková et al., 2020^81^
Pompe disease	39	Pelvic girdle muscle weakness, respiratory insufficiency	49	*GAA* (NM_000152.3)	IVS1‐13 T > G (85.3%)	Spain	Alonso‐Pérez et al., 2020^82^
			1	*GAA* (NM_000152.3)	c.546G > T	Japan	Hossain et al., 2020^83^
			126	*GAA* (NM_000152.3)	c.‐32‐13 T > G (90%)	France	Laforet et al., 2013^46^
			54	*GAA* (NM_000152.3)	Not available	Netherlands	Hagemans et al., 2005^84^
Mucolipidosis III	44	Osteoarthritis, scoliosis, hypoplasia, retinitis pigmentosa	6	*GNPTAB* (NM_024312.4)	No common mutation reported	Netherlands	Oussoren et al., 2018^37^
			1	*GNPTAB* (NM_024312.4)	IVS7—1G > A	USA	Steet et al., 2005^85^
Niemann‐pick type C	41	Gait disturbance, difficulty in speech, cognitive decline, ataxia, cerebral and cerebellar atrophy, vertical supranuclear ophthalmoplegia	2	*NPC1* (NM_000271.4)	p.Arg934Ter; p.Pro471Leu	USA	Vo et al., 2022^86^
			1	*NPC1* (NM_000271.4)	p.A518T /p.A1059G	Korea	Kim et al., 2020^87^
			1	*NPC1* (NM_000271.4)	p.P474L	Germany	Piroth et al., 2017^88^
			13	*NPC1* (NM_000271.4)	P1007A (38%)	France	Sevin et al., 2007^89^
Type 1 Sialidosis	25	Myoclonus, ataxia, epilepsy, visual disturbances	18	*NEU1* (NM_000434.4)	c.544A > G (91.7%)	Taiwan	Hu et al., 2018; Lai et al., 2009^50^
			12	*NEU1* (NM_000434.4)	c.239C > T (50%)	Mainland China	Lv et al., 2020^90^

Overall, the clinical presentation noted in adult Gaucher, Fabry and Pompe patients from our cohort were hepatosplenomegaly, angiokeratoma and progressive muscle weakness, respectively. This is in congruence to that observed by other groups.^11^, ^12^, ^34^ Likewise, we observed previously reported common clinical features namely short stature, mild coarse facial features, kyphoscoliosis, pectus carinatum in our adult MPS IVA patients,^35^ while in adult MPS I patients, hernia, joint contractures and corneal clouding were seen, which is in concordance with the earlier reported cases.^36^ In cases of adults with mucolipidosis II/III, and Niemann–Pick disease type B, the clinical signs observed were similar to that described in the literature.^37^, ^38^ Importantly, presence of myoclonus and ataxia are the key signs in adult sialidosis patients,^39^ which was also observed in both our patients. Recently, several adult patients with gait ataxia, early signs of dementia, white matter abnormality and psychiatric issues^40^ are being identified with MLD; however, MLD patients in our cohort showed gait ataxia and bilateral tremors as the key signs. Nijmeijer et al. reported psychiatric issues such as attention deficit hyperactivity disorder (ADHD) and aggressiveness as the common signs^13^ in adult MPS‐III patients. In this study, we could not identify adult MPS‐III patients, this is likely because majority of these patients are being referred to psychiatric clinics^13^ and increased awareness among the psychiatrists will likely aid in their early detection.

In view of the genotype data seen in our adult LSD patients, the variant p.Leu483Pro in the *GBA1* gene, which has been reported in 60% of Gaucher patients in India^15^ was seen in 23% of the cases in the present adult cohort. Of note, this variant was seen in compound heterozygous state with another pathogenic variant on the other allele. Similar observation has been made in adult Gaucher patients where the authors propose that a milder phenotype of adult GD could be attributed to the presence of missense variant other than p.Leu483Pro on the second allele.^41^ Surprisingly, variant Asn409Ser in the *GBA1* gene has been the most common variant reported in ~80% of adult Gaucher patients^34^ but was not observed in our study. This suggests genetic heterogeneity in the Indian Gaucher patients. For adult Fabry patients, no common variant was identified in our study. Furthermore, two variants, N215S and F113L, that are commonly reported in the *GLA* gene in adult Fabry patients^42^, ^43^ were not identified in this cohort. The variant p.R363H identified in patient P20 has been previously reported in an Indian Fabry patient^44^ and is mostly associated with the late‐onset phenotype,^45^ which is similar to our observation. Of note, in adult‐onset Pompe patients, the variant −32‐13 T > G in the *GAA* gene is the most common variant reported and is seen in at least one allele in 90% of these patients.^46^ Surprisingly, previous study on late‐onset Pompe patients in India as well as in our patient, we did not find this common variant.^17^ Furthermore, as this variant has been seen in asymptomatic patients as well as in patients with severe phenotype,^46^ it is difficult to ascertain genotype–phenotype correlation. In our study, we identified a missense variant c.1841C > T in the *GAA* gene, which has been reported previously in late‐onset Pompe patients in a compound heterozygous state and is likely to have mild phenotype.^47^ However, due to lack of variant details in other patients, it is difficult to determine a genotype–phenotype correlation.

We found a previously reported founder variant p.P77R in the *GALNS* gene in an adult MPS‐IVA patient. This variant has been associated with a severe phenotype^48^; however, due to lack of clinical details and patient being lost to follow‐up, it is not possible to comment on the delayed clinical presentation. Previously, in attenuated MPS I patients, the variant p.L490P has been reported as one of the most common genotypes (21/158; 13.3%),^49^ which was also seen in our patient P17. Study in Taiwan showed that in type I sialidosis patients, the variant c.544A > G in the *NEU1* gene is the most common variant seen in 91.7% cases,^50^ but this variant was not observed in our patients. Remarkably, one of our type I sialidosis patients had a novel splice site variant *NEU1*: c.1021 + 4A > T reported for the first time, whereas in another patient, a novel missense variant along with a previously reported variant p.G243R was seen in likely compound heterozygous state. The variant p.G243R has also been seen previously in Type 1 sialidosis patient.^51^ In adult MLD patients with a slower progression of the neurological manifestations, the variant I179S in the *ARSA* gene has been commonly seen.^52^ However, genetic study was not performed in our adult MLD patient to comment on the same. Interestingly, we found a single adult patient of MLD due to Sap‐B deficiency and this is the third report in the world.^32^ In adult NPD‐B patients, recent study by Lidove et al. showed p.Arg610del in the *SMPD1* gene in 62% of the cases suggesting it to be associated with attenuated phenotype. This variant was not seen in our patients, rather, we identified a variant c.1693G > T in the *SMPD1* gene in one of our adult NPD‐B patient, which has previously been associated with mild phenotype.^53^

Considering the heterogeneity in the clinical presentation as well as variant spectrum for LSDs in adult patients, it is difficult to ascertain a genotype–phenotype correlation. Nonetheless, few studies on biochemical and structural characterisation of certain missense variants in the LSD genes like *GALNS* and *SMPD1* have shown a high residual activity of the respective enzymes,^48^, ^53^, ^54^ particularly in case of adult presentations. Likewise, in two of our cases with novel missense variant in the *IDUA* and *GLA* gene, we used in‐silico tool and found that there was no major structural change detected in the resultant mutant protein. Thus, a high residual enzyme activity may be one of the reasons for the adult presentation in these cases. With more functional studies in future, it will be possible to decipher the underlying molecular basis in adult patients with LSDs.

## 5 CONCLUSION

Overall, this study describes LSDs in an adult population from a tertiary genetic centre in India for the first time. Our cohort shows Gaucher disease to be the most common adult LSD followed by Fabry disease, MPS IVA, MPS I, Pompe, NPD‐B, MLD, Sialidosis and ML‐III. However, adult LSDs like MPS‐III, Tay‐Sachs disease and Krabbe disease were not identified in the present cohort, likely because of poor awareness of presentation of these conditions among the adult neurologists and psychiatrists. Interestingly, the molecular spectrum observed in Gaucher and Fabry patients in the present cohort is different as compared to findings in other populations, suggesting genetic heterogeneity. The clinical pointers in these patients were mild hepatosplenomegaly, angiokeratoma, muscle weakness, short stature with skeletal dysplasia, gait imbalance with tremors, myoclonus and cherry red spot. With more cases reported in future, there will be a better understanding on this group of single gene disorders, in order to provide genetic counselling, prognosis and timely treatment to the affected families.

## AUTHOR CONTRIBUTIONS

*Conceived and designed experiments*: Jayesh Sheth, Aadhira Nair and Harsh Sheth. *Patient recruitment and clinical analysis*: Jayesh Sheth, Koumudi Godbole, Chaitanya Datar, Sheela Nampoothiri, Inusha Panigrahi, Heli Shah, Shruti Bajaj, Naresh Tayade, Naveen Bhardwaj. *Enzyme study*: Riddhi Bhavsar. *Sequencing data analysis and interpretation*: Harsh Sheth and Aadhira Nair. *Writing first draft of the manuscript*: Aadhira Nair and Jayesh Sheth. *Made critical revisions and approved final version*: Jayesh Sheth and Harsh Sheth. All authors reviewed and approved the final manuscript.

## FUNDING INFORMATION

We sincerely acknowledge the research grant from the Department of Biotechnology, Government of India, DBT BT/PR39587/MED/12/851/2020 and Gujarat State Biotech Mission, Department of Science and Technology (GSBTM‐DST/JDR D/608/2020/459‐461) for this work.

## CONFLICT OF INTEREST STATEMENT

The authors declare no conflicts of interest.

## INFORMED CONSENT

All procedures followed were in accordance with the ethical standards of the institutional ethics committee of FRIGE's Institute of Human Genetics (Reg No‐E/13237) and with the Helsinki Declaration of 1975, as revised in 2000. Informed consent was obtained from all patients for being included in the study.

## ANIMAL RIGHTS

This article does not contain any studies with human or animal subjects performed by any of the authors.

## Supporting information


**DATA S1.** Supporting Information.

## Data Availability

Not applicable.
