# Supplementary material for: Lysosomal storage disorders identified in adult population from India: Experience of a tertiary genetic centre and review of literature
Source: JIMD Rep. 2024 Jan 2;65(2):85–101. doi: 10.1002/jmd2.12407 (PMC10910243; doi:10.1002/jmd2.12407)
Supplement: Supplementary file 1 — DATA S1. Supporting Information. [file JMD2-65-85-s001.docx]

Table 1: List of primers used in Sanger sequencing and for PCR-RFLP

| **Gene/Exon** | **Primer sequence FP** | **Primer sequence RP** |
| --- | --- | --- |
| GBA/ Ex 1 | CCTAAAGTTGTCACCCATAC | CAACCCTTCTGATGACAACT |
| GBA/ Ex 2 | GGAGAGGGGCTTGCTTTTCA | GGAGGCAGAGGTTGGAATGA |
| GBA/ Ex 3-4 | CAAGGGGTGAGGAATTTTGA | CACCACTGCACTCCTGTCTC |
| GBA/ Ex 5-6 | TGGCCCTGACTCAGACACTA | CTGATGGAGTGGGCAAGATT |
| GBA/ Ex 7 | GGCTGTTCTCGAACTCCTGA | ATAGTTGGGTAGAGAAATCG |
| GBA/ Ex 8 | AGTTGCATTCTTCCCGTCAC | ATCATGGTTCCCCAGAGTTG |
| GBA/ Ex 9 | CAGCTGCCTCTCCCACAT | GTGTGCCTCTTCCGAGGTT |
| GBA/ Ex 10-11 | GAGAGCCAGGGCAGAGCCTC | CTCTTTAGTCACAGACAGCG |
| GBA/ for L483P | CTGAACCCCGAAGGAGGACC | GGGCTTACGTCGCTGTAAGCTCACAC**C**GGC |
| NEU1/ Ex 1 | GGCTTAAGGGTGACATCTGC | GGGGTCTGGGAGAAAGAAAA |
| NEU1/ Ex 2 | CCCCATTAGATCCTCCCTCT | CTCAGGCAACCAACCCTCTA |
| NEU1/ Ex 3 | CCTGAGGTCCCTAGCAGAAG | AATCTTCCCCTTGGAAAGGA |
| NEU1/ Ex 4 | GATTGCATTTGGGAAGTGGT | AAGGGTGTGTGGCACTGAG |
| NEU1/ Ex 5-6 | CTGCCCTCCAGAACATCATT | ACTGTCTTTCAGGCGTCTCC |
